# Active Fixation Atrial Pacemaker Lead Placement With a Modified Guiding Catheter in Persistent Left Superior Vena Cava: A Rare Case Report

**DOI:** 10.1002/joa3.70178

**Published:** 2025-08-19

**Authors:** Junichi Sugiura, Taku Nishida, Tomoko Kikawa, Kenichi Ishigami

**Affiliations:** ^1^ Cardiovascular Medicine Nara City Hospital Nara City Japan; ^2^ Department of Cardiovascular Medicine Nara Medical University Kashihara City Japan

**Keywords:** active fixation lead, atrial pacemaker lead fixation, pacemaker implantation, persistent left superior vena cava

## Abstract

The active fixation atrial pacemaker lead was successfully placed in the proximal part of the persistent left superior vena cava via a retrograde approach with a J‐shaped stylet and the modified guiding catheter, which was cut proximally by approximately 10 cm and held at the edge with forceps.

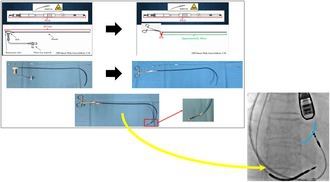

Persistent left superior vena cava (PLSVC) is a rare anatomical anomaly found in 0.47% of patients undergoing cardiac implantable electronic device implantation [1]. This anomaly may complicate pacemaker implantation; for example, access to the right atrium or ventricle may become challenging. Active fixation atrial pacemaker leads are generally placed in the right atrial appendage (RAA), septum (RAS), or free wall (RAFW). However, these sites may be inappropriate because of their low P‐wave amplitudes or high thresholds. Herein, we report a challenging case involving the difficult placement of an active fixation atrial pacing lead at these sites, with successful atrial lead implantation into the PLSVC.

A 77‐year‐old woman had undergone pulmonary vein and PLSVC isolation for paroxysmal atrial fibrillation and was admitted to our hospital with syncope. Ambulatory electrocardiography (ECG) monitoring revealed sustained ventricular tachycardia (VT), requiring catheter ablation. However, no intervention was performed for VT, as we could not induce VT and identify its arrhythmogenic substrates. Although the VT was treated with amiodarone, amiodarone caused sick sinus syndrome (SSS) and consequent heart failure. A 12‐lead ECG revealed a junctional rhythm with a heart rate of 45 beats/min, while echocardiography revealed no specific abnormal findings except for the presence of PLSVC. The patient subsequently underwent implantable cardioverter defibrillator (ICD) implantation for VT and SSS.

ICD implantation was performed on the right side due to the presence of a PLSVC, during which shock lead (7122Q‐58 cm, Abbott, U.S.) was placed at the right ventricular apex. We attempted to place an active fixation atrial pacing lead (2088TC‐46 cm, Abbott, U.S.) in the RAA and RAS; however, the P‐wave amplitude was low, and the threshold was high at these sites. Therefore, the atrial lead was placed in the RAFW (P‐wave amplitude = 0.9 mV; pacing threshold = 1.0 V at 0.5 ms; impedance = 388 Ω). However, echocardiography performed 2 days following ICD implantation revealed pericardial effusion, and pericardial drainage was performed. Following pericardial drainage, monitoring ECG revealed pacing failure of both the atrial and shock leads (atrial/ventricular pacing threshold = 5.25 V at 0.5 ms/4.75 V at 0.5 ms). Nonetheless, chest radiography performed before and after pericardial drainage revealed no dislodgement of either lead. Because chest plane computed tomography revealed atrial lead perforation, ICD reimplantation was performed with cardiac surgical backup.

Both leads were successfully removed without exacerbating pericardial effusion. A shock lead was subsequently implanted into the apical ventricular septum. Similar to the initial ICD implantation, the P‐wave amplitude was low, and the threshold was high in both the RAA and RAS, whereas a high P‐wave amplitude (over 2 mV) without far‐field ventricular wave sensing was detected on the left atrial (i.e., roof) side of the coronary sinus (CS) and PLSVC. Although we attempted lead fixation at these sites with a J‐shaped stylet, it was challenging because the lead screw was nearly parallel to the wall of the PLSVC (Video S1). A guiding catheter (GC) for CS (CPS Direct Wide Curve, Abbott, U.S.) was applied to approach the PLSVC and contact the active fixation lead perpendicular to the PLSVC wall. Because this GC was longer than the pacing lead (50.7 and 46 cm, respectively), we cut the GC proximally by approximately 10 cm and held the edge with forceps to control it (Figure 1). After exchanging the 7‐Fr sheath introducer to the modified GC (mGC) directly over a wire, the atrial lead was introduced via the mGC using a J‐shaped stylet. The lead screw was subsequently directed perpendicularly to the PLSVC and successfully placed in the proximal part of the PLSVC (Video S2). After slitting and cutting the mGC with ophthalmological scissors, the atrial lead was fixed adequately (Figure 2). Each parameter of atrial and shock lead was verified as appropriate (P‐wave amplitude = 2.1 mV; pacing threshold = 0.5 V at 0.5 ms; impedance = 360 Ω for atrial lead; R‐wave amplitude > 12.0 mV, pacing threshold = 0.5 V at 0.5 ms and impedance = 340 Ω for shock lead); however, the current injury could not be detected.

**FIGURE 1:**
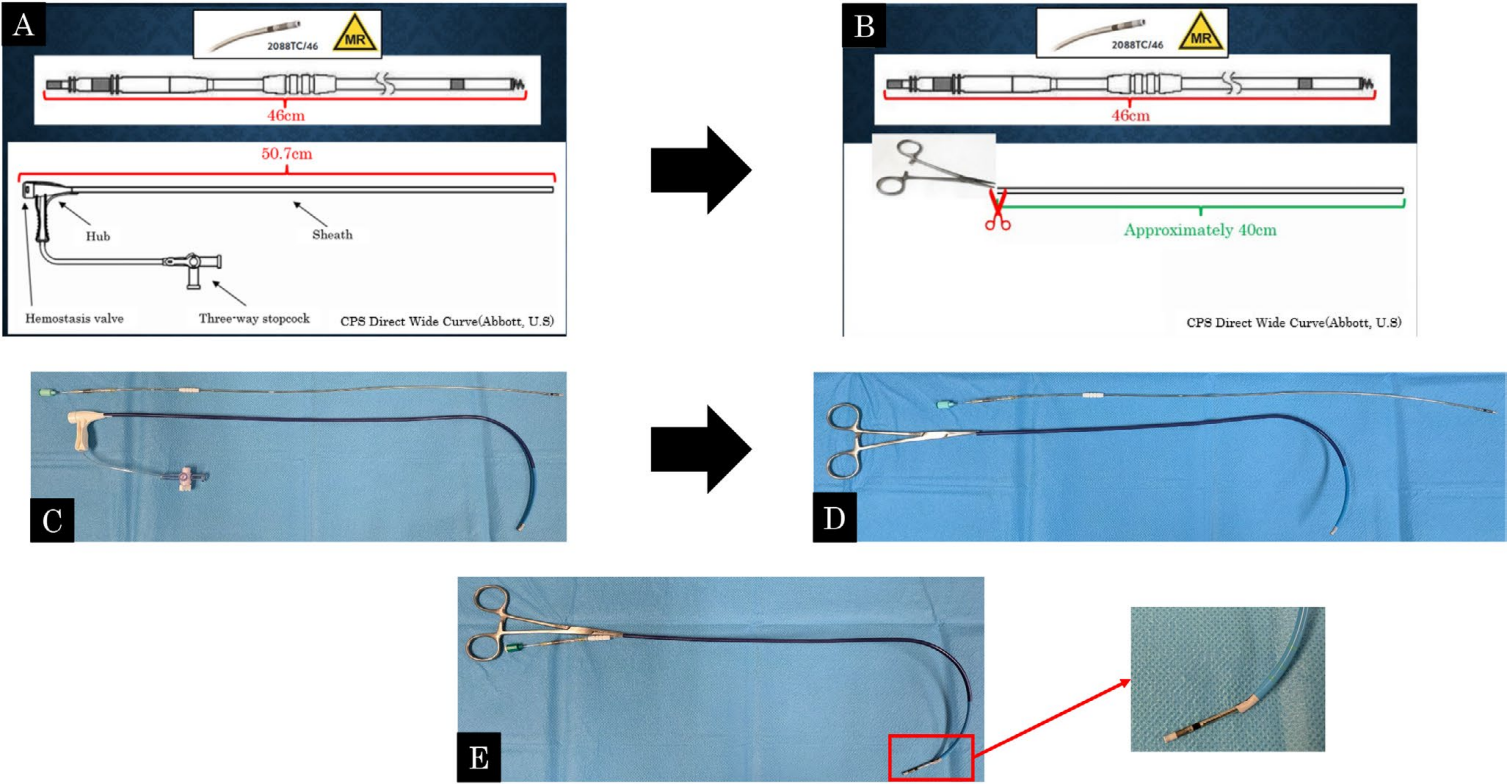
(A) The Tendril STS 2088TC/46 (Abbott, U.S.) and CPS Direct Wide Curve (Abbott, U.S.). (B) Modified CPS Direct Wide Curve cut off proximally by approximately 10 cm with the edge held with forceps. (C) A picture of the Tendril STS 2088TC/46 and CPS Direct Wide Curve. (D) A picture of the Tendril STS 2088TC/46 and modified CPS Direct Wide Curve with the secure edge held with forceps. (E) A picture of the Tendril STS 2088TC/46 introduced via a modified CPS Direct Wide Curve.

**FIGURE 2:**
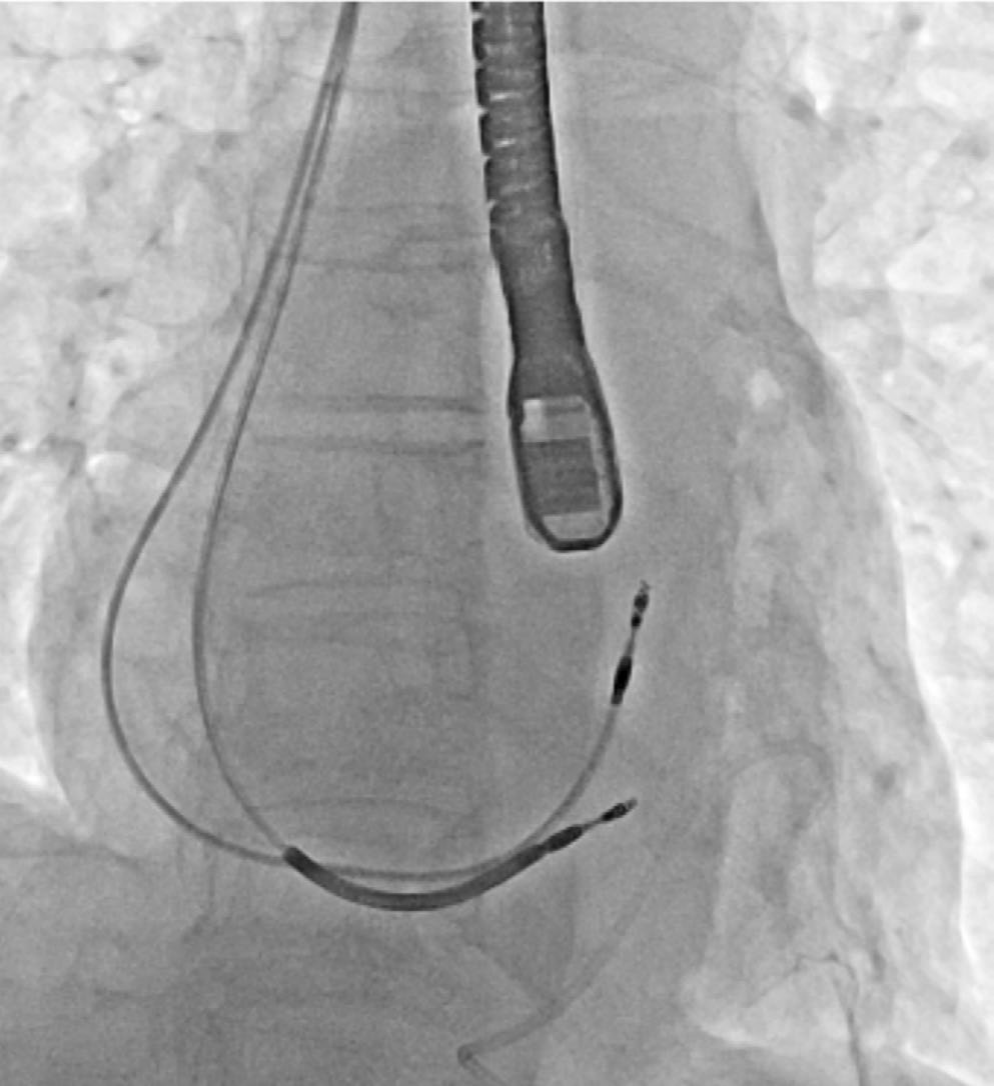
Chest radiograph after slitting the guiding catheter showing that the atrial lead was fixed adequately.

Chest radiography and ECG following ICD reimplantation demonstrated neither lead dislodgement nor pacing failure of the atrial lead. All atrial lead parameters remained stable at the 10‐month follow‐up (P‐wave amplitude = 3.3 mV; pacing threshold = 1.25 V at 0.5 ms; impedance = 400 Ω).

PLSVC is a rare congenital anomaly for which various lead placement strategies have been reported, including atrial lead implantation in the lateral wall of the right atrium via PLSVC using the left subclavian approach [2]; passive fixation lead placement at the right ventricular apex via PLSVC using the right subclavian approach [3]; and left ventricular lead insertion into the lateral coronary vein on the right side via PLSVC [4].

A previous study evaluated permanent left atrial pacing by implanting a pacing lead into the CS [5]. However, the clinical superiority of pacing from the CS over right atrium pacing was not demonstrated herein. Thus, pacing from the CS might be preferable only in cases of poor acute sensing and pacing thresholds at multiple locations within the RA, such as atrial standstill.

In this case, a 46 cm Tendril lead was reused; therefore, mGC was needed to replace the lead in PLSVC. If a longer lead is selected when a lead is replaced with PLSVC, mGC is not necessary.

Our case presented several novel aspects. First, the isolated PLSVC resulted in a high P‐wave amplitude detectable only proximally. Second, an mGC enabled perpendicular contact with the PLSVC wall. The mGC and J‐shaped stylets facilitated successful active fixation lead placement. Third, an atrial lead was inserted retrograde. Fourth, PLSVC lead placement represents a novel approach. While PLSVC lead fixation risks cardiac tamponade or far‐field ventricular sensing, placement was completed without complications. Further clinical experience remains necessary for safe PLSVC lead placement, as long‐term lead parameter stability remains challenging.

To the best of our knowledge, this is the first reported case where an active fixation atrial pacemaker lead is implanted into an isolated PLSVC via a retrograde approach for PLSVC with mGC.

## Disclosure

Approval of the research protocol: Case reports do not require approval at our institute.

## Consent

The authors have nothing to report.

## Conflicts of Interest

The authors declare no conflicts of interest.

## Supporting information


**Video S1:** The atrial lead screw is nearly parallel to the wall of the PLSVC and slips into the distal PLSVC during fixation into the wall of the PLSVC with only a J‐shaped stylet. The blue lines represent the walls of the CS and PLSVC. CS, coronary sinus; PLSVC, persistent left superior vena cava.
**Video S2:** Using the modified guiding catheter and J‐shaped stylet, the atrial lead screw was directed perpendicularly to the PLSVC and successfully fixed in the proximal part of the PLSVC. The blue line represents the walls of CS and PLSVC. CS, coronary sinus; PLSVC, persistent left superior vena cava.

## Data Availability

Data will be made available at a reasonable request from the authors.
