# Supplementary figures and images for: Active Fixation Atrial Pacemaker Lead Placement With a Modified Guiding Catheter in Persistent Left Superior Vena Cava: A Rare Case Report
Source: J Arrhythm. 2025 Aug 19;41(4):e70178. doi: 10.1002/joa3.70178 (PMC12362294; doi:10.1002/joa3.70178)

## Slide 1
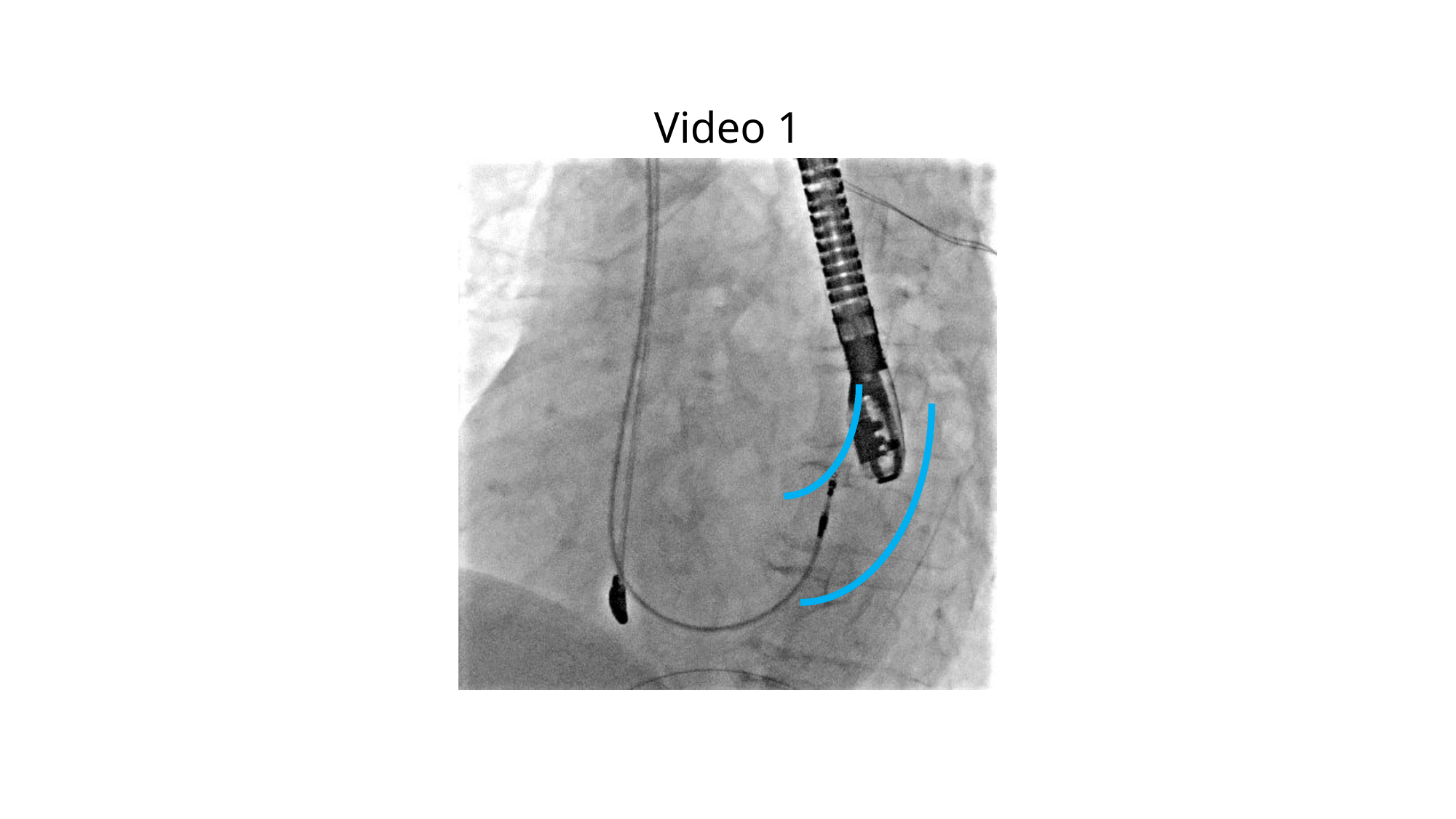

Video 1

## Slide 2
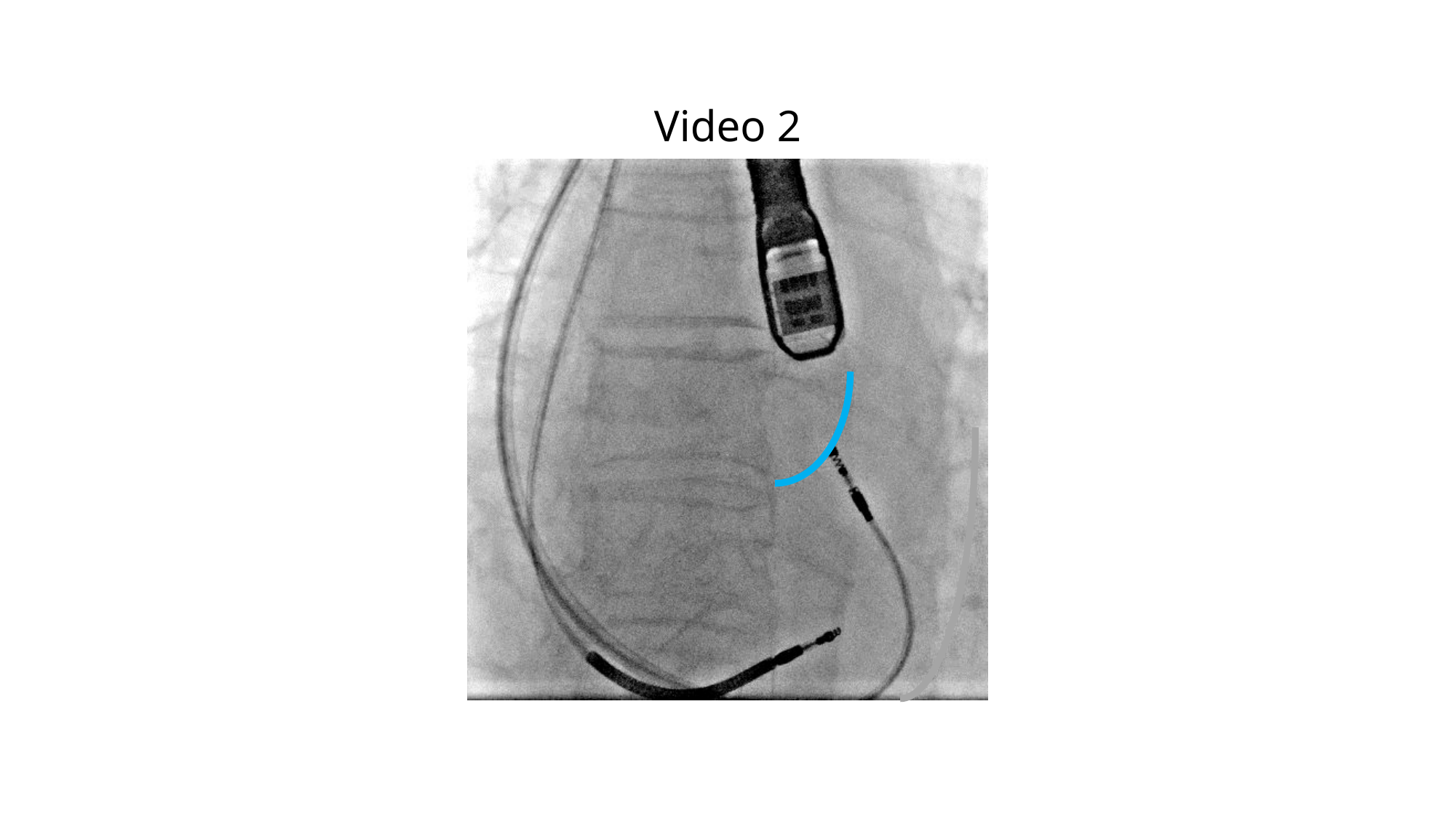

Video 2

Supplement: Supplementary file 1 — Video S1: The atrial lead screw is nearly parallel to the wall of the PLSVC and slips into the distal PLSVC during fixation into the wall of the PLSVC with only a J‐shaped stylet. The blue lines represent the walls of the CS and PLSVC. CS, coronary sinus; PLSVC, persistent left superior vena cava. Video S2: Using the modified guiding catheter and J‐shaped stylet, the atrial lead screw was directed perpendicularly to the PLSVC and successfully fixed in the proximal part of the PLSVC. The blue line represents the walls of CS and PLSVC. CS, coronary sinus; PLSVC, persistent left superior vena cava. [file JOA3-41-e70178-s001.pptx]
